# Flexible Work and Organizational Commitment Among Korean Managers: The Mediating Role of Work–Family Conflict and CEO Gender Equality

**DOI:** 10.3390/bs15101406

**Published:** 2025-10-16

**Authors:** Hyondong Kim, Jin Suk Lee

**Affiliations:** Dongguk Business School, Dongguk University-Seoul, Seoul 04620, Republic of Korea; kim1415@dongguk.edu

**Keywords:** flexible work arrangements (FWAs), work–family conflict, CEO gender equality perceptions, organizational commitment, gender roles

## Abstract

This study aims to explore how organizations plan and implement flexible work arrangements (FWAs) to support managers in fostering work–family balance. In doing so, we examine the sequential mediating roles of work–family conflict, CEO gender equality perceptions, and organizational commitment to elucidate the consequences of FWAs. Our study draws upon the Korean Women Manager Panel (KWMP), a three-year initiative that includes 2345 mother and father managers working in 469 Korean companies. We utilized the longitudinal multilevel macro process model 8 to examine the mediating effects of work–family conflict and CEO gender equality perceptions on the relationship between FWAs and organizational commitment. The findings show that both work–family conflict and CEO gender equality perceptions mediate the relationship between FWAs and organizational commitment. Notably, father managers perceive less work–family conflict than mother managers, which indicates that as FWAs increase, CEO gender equality perceptions and organizational commitment rise as well. The use of FWAs is more beneficial for father managers as it alleviates work–family conflict and fosters positive perceptions and attitudes about CEOs and organizations. Thus, to increase the effectiveness of FWAs, it is pivotal to consider managers’ gender. Additionally, the CEO must be actively involved in shaping and promoting gender equality in the workplace.

## 1. Introduction

In South Korea, participation of females and dual-earning couples in the workforce has been increasing over recent decades (Hoobler et al., 2009; Ongaki, 2019; Statistics Korea, 2024). Gender statistics collected, analyzed, and published by the Korea Women’s Development Institute reveals a share of female managers around 21~22%, which is estimated far lower than OECD countries’ average share of female managers (33.7%) (The Korea Herald, 2023; Han et al., 2025). Thereby, it is critical to explore how Korea society supports female managers to stay in the labor market and develop career tracks to promote them into taking professional and managerial positions.

Gender stereotyping that presumes a separation between work and family by gender is a traditional cultural norm in Korean society. These gendered norms pressure mother managers to prioritize family roles and responsibilities over their job (Hoobler et al., 2009; Ongaki, 2019; Oh & Mun, 2022). In particular, female Korean managers face more challenges in devoting time, attention, and energy to achieving job performance, as mother managers are presumed to assign more importance to family than to their job (Shockley et al., 2021; Oseghale et al., 2024). To help address work–family conflict in Korean society, flexible work arrangements (FWAs) have been designed and implemented to provide more choice over working spaces and schedules (T. D. Allen et al., 2013; Adams & Schwarz, 2024). Thereby, Korean corporations provide FWAs for company managers and employees to adjust their times and locations to respond to conflicting interplays among work, family, and personal life domains. According to Statistic Korea in 2025, the use of FWAs was estimated to be around 4~5% in 2015–2017 (Statistics Korea, 2025). After 2017, the use of FWAs increased by 8~10% (Statistics Korea, 2025). After the COVID-19 pandemic, the use of FWAs is estimated to be 14.2% in 2020, 16% in 2021–2022, and 15% in 2023–2024 (Statistics Korea, 2025). The existing literature has highlighted the potential benefits and detriments of FWAs in work–family relations: while they can alleviate work–family conflict, multitasking between work and family domains can also exacerbate inter-role pressures (T. D. Allen et al., 2013; Singh et al., 2018; Bayazit & Bayazit, 2019; Kossek et al., 2023). This current study tests the extent to which FWAs provide employees discretion over their allocation of energy, attention, and time resources into work and family, which can reduce the strain associated with work–family conflicts.

Moreover, employees’ perceptions of CEO gender equality are presumed to intervene in the effects of work–family conflict on organizational commitment. CEO gender equality perceptions refer to the extent to which employees believe their CEO prioritizes gender equality when shaping and developing human resource management (HRM) practices and programs (Joo et al., 2024). Higher levels of CEO gender equality perceptions can help alleviate the stereotyping of female workers, which highlights the importance of family roles as well as work roles. CEOs who prioritize gender equality can create an environment that enables employees to effectively balance their work and family responsibilities. Thus, CEO gender equality perceptions can enhance organizational commitment by shaping and developing employees’ beliefs that the organization will help them address conflicting demands related to work and family.

Furthermore, family roles and demands are perceived as more salient for working mothers, as social pressures and gender stereotyping urge female employees to prioritize family, and they tend to be more involved in family matters (Shao et al., 2021; Oseghale et al., 2024). Working mothers often perceive more interference between work and family (Hoobler et al., 2009); therefore, gender may determine the activation of family identity, which significantly affects the level of perceived work–family conflict. Notably, there may be a significant gender difference in employees’ perceptions of CEO gender equality and organizational commitment, which are closely associated with work–family conflict levels.

This study contributes to the literature on FWAs and organizational commitment through a longitudinal study based on three years of data, identifying how and when FWAs are effective. Specifically, it investigates the roles of work–family conflict and CEO gender equality perceptions as underlying mechanisms. Additionally, it reveals a distinct gender difference, setting it apart from previous studies: FWAs are more effective for male managers than female ones. These findings have managerial implications for HR practitioners in implementing and utilizing FWAs within organizations. Specifically, it is not just about implementing FWAs into an organization; it is about setting the climate for managers to actually use them.

## 2. Literature Review and Hypothesis Development

### 2.1. FWAs and Work–Family Conflict

Previous studies have suggested that FWAs support managers who are parents in resolving the challenges associated with work–family conflict (von Allmen et al., 2024). Shockley et al. (2021) examine dual-earning couples’ strategies to respond to the COVID-19 lockdown. One third of dual-earning samples rely on mothers to perform childcare, which negatively affect mothers’ well-being and job performance. Shao et al. (2021) show that work–family boundary stressors (e.g., mutual interference between work and family) and work coordination stressors (communication activities with coworkers) are associated with the choice of FWAs. Gašić and Berber (2023) showed that the implementation of FWAs provides autonomy and control over jobs, which enables employees to balance work and family domains and engage more in their job tasks. The statistical results of Jia et al.’s (2024) study reveal that job-related factors such as job resources and job demands are antecedents of FWA utilization. Job resources provide more choices for employees to use FWAs, and job demands cause employees to utilize FWAs to buffer its negative effects. Haines et al.’s (2024) study shows that FWA implementation increases job control and work engagement, which results in lower turnover. In Toscano and Zappalá’s (2024) study, when company workers have good job performance, this is likely to increase with FWAs, which results in higher productivity in the use of FWAs. Previous studies suggest that time, attention, and energy are finite resources that must compete in these domains, which exacerbates conflicting work–family relations (von Allmen et al., 2024). FWAs are considered to offer employees control over organizing and negotiating work–family boundaries (T. D. Allen et al., 2013; von Allmen et al., 2024), as parents can use them to adjust the scope of work and family domains. FWAs blur the demarcation of work and family domains, increasing self-control over schedules, locations, and procedures for carrying out work tasks (T. D. Allen et al., 2013; Ongaki, 2019; Kumar et al., 2023). They also enhance the physical and psychological well-being of managers who are also parents, helping to balance conflicting interests. Thereby, implementing FWAs can positively influence parent managers’ control over the conflicts caused by fulfilling work and family roles.

**Hypothesis 1.**  *FWAs negatively influence work–family conflicts.*

### 2.2. FWAs, Work–Family Conflict, CEO Gender Equality Perception, and Organizational Commitment

Work–family conflict can become exacerbated when the roles and demands between work and family domains are incompatible. As the conflict intensifies, it can drain the time and energy available for performing work and family roles (Haines et al., 2024; Oseghale et al., 2024). As this conflict increases, the resulting multitasking imposes role pressures. As work and family interfere with each other, organizational environments are perceived as obstructing the work and family role requirements. Traditionally, family roles and responsibilities are often seen as more salient for mothers. Thus, high levels of work–family conflict disadvantage mother managers more than fathers and can increase their stressful experiences (Gajendran et al., 2024; Jia et al., 2024). When managers struggle to simultaneously satisfy work and family demands, they may perceive that organizational policies and programs do not adequately support work–family balance. As mothers often assign more importance to family matters and responsibilities, this lack of support can be viewed as hindering gender equality. Because mother managers are stereotyped as devoting more time and attention to their family, their performance may be perceived as less than that of father managers, as organizations perceive fathers to be more compatible with workplace leadership requirements, causing organizations to be considered as less supportive of gender equality (Cha & Kim, 2023; Oh & Mun, 2022). Thereby, high levels of work–family conflict exacerbate negative perceptions of CEO gender equality.

Flexibility, enhanced through FWAs, allows managers to negotiate work–family boundaries (Kossek et al., 2023). FWAs help managers manage family matters and responsibilities, as work–family boundaries are blurred to facilitate transitions between work and home (Adams & Schwarz, 2024). In particular, FWAs are more beneficial for managers as they can devote resources to satisfying family roles and demands. CEOs exert decision-making authority to influence the adoption and establishment of FWAs (Vadvilavicius & Stelmokiene, 2024), which can be interpreted as their intentions to support work–family balance (Joo et al., 2024). FWAs can mitigate the negative effects of work–family conflict on employees’ perceptions of a CEO’s gender equality philosophy by signaling that the CEO prioritizes work–family balance. Few studies have been conducted to examine the importance of the CEO’s role in shaping and promoting gender equality working environments. Several studies have shown that a gender equality policy is moderately effective to support female workers’ career development. The statistical results of Pas et al.’s (2014) study reveal that gender equality arrangements slightly increase women physicians’ career motivations and support those women physicians prioritizing roles between career and family. Ojwala et al.’s (2024) study demonstrates that gender equality policies and staff members’ assigned gender equality role ameliorate gender discriminatory effects against female workers’ career progression. Kocani et al.’s (2025) study shows that lack of gender equality policies reinforces gendered norms and cultural barriers against female workers’ career struggles in male-dominated workplaces. Gender equality policy is pivotal to addressing normative pressures and cultural biases stem from gender stereotypes entrenched in the workplace. CEO exerts authority over initiating, building, and developing gender equality policy, one of which is FWAs aim at alleviating stress from work–family conflicting relations for female managers (Wang & Cheng, 2025). Thereby, CEO gender equality policy serves as links among FWAs, work–family conflict, and attitudes toward organization. When employees view CEOs as caring, they are more likely to reciprocate with positive attitudes toward the organization (Wayne et al., 2013). Similarly, managers are likelier to commit to an organization in exchange for the CEO’s support of work–family balance through FWA implementation (Sublet et al., 2024). FWA policies provide employees discretion over work schedules and workspaces, which enhances employee motivations and abilities to demonstrate innovative behaviors (Azeem & Kotey, 2023). Innovative behaviors allow employees to make contributions to organizational competitiveness by creating and developing new knowledge and applying it to improving product and service functions and features (Jiang et al., 2023). Shifrin and Michel (2022) conducted meta-analysis to examine the effects of FWAs on employee work behaviors and physical health. Meta-analysis by Shifrin and Michel (2022) demonstrated that implementing FWAs motivates employees to engage in exercise and yoga and to participate in workplace activities. FWAs make positive consequences for company managers such as mitigating work–family conflict, facilitating innovative behaviors, and promoting physical health. FWAs can enhance organizational commitment by highlighting benefits from FWA implementation. Thereby, FWAs foster positive perceptions of CEO gender equality and organizational commitment by reducing work–family conflict.

**Hypothesis 2.**  *FWAs promote CEO gender equality perceptions and organizational commitment through reduced work–family conflict.*

### 2.3. Gender Roles

The existing literature has suggested that FWAs are more effective for mother managers in promoting work–family balance (Wayne et al., 2013; Ongaki, 2019). As FWAs increase the permeability and flexibility of temporal, physical, and psychological work–family boundaries, female workers can utilize them to address gendered assumptions about family obligations. However, contrary to previous studies on FWAs and gender, this current study assumes that FWAs are more beneficial in supporting the work–family relations of fathers who are managers. Gendered assumptions in the workplace suggest that working fathers prioritize their job, while working mothers must commit to family care responsibilities (Oh & Mun, 2022). Existing studies on working mothers have revealed that female managers tend to engage in a larger share of housework and childcare, which prevents them from fully committing to their job (Cha & Kim, 2023). Brega et al. (2023) demonstrated that gendered assumptions about work and family relations are salient for working women in countries where gender differences do not result in availability and accessibility for FWA policies. The statistical results of Erdogan et al.’s (2021) study revealed that a high level of salience in work and family roles accelerated the level of work–family conflict. The mothers sampled in this study work in professional and managerial roles. For mothers who hold positions and develop careers in professional and managerial fields, family roles are significantly salient to their personal identities, as they struggle to satisfy work and family demands simultaneously with a limited amount of resources (Erdogan et al., 2021). Working mothers in these positions tend to be highly committed to their career tracks (Cha & Kim, 2023; Shin & Kim, 2023). While their jobs may be demanding, they often prioritize the challenges associated with household labor and childcare responsibilities. Mother managers are often stereotyped as devoting resources to the family domain by avoiding difficult tasks and long work hours (Shin & Kim, 2023). When they utilize FWAs to mitigate work–family conflicts, normative assumptions encourage them to prioritize family over work. However, as mothers in professional and managerial positions often strive for career success, they must juggle the transition between home obligations and work commitments (Cha & Kim, 2023; Oh & Mun, 2022). Working mothers in these positions may fear that FWAs stigmatize them by suggesting they prioritize their family over their job at the expense of their career (Cha & Kim, 2023; Shin & Kim, 2023). As family role is salient to mother managers, mother managers experience interference from their work role with their family role. FWAs are deemed tools for mother managers to meet mothering expectations. Mother managers are prone to work–family conflict, which is escalated when they prioritize family care over company work, especially when FWAs provide additional resources that allow them to allocate more time to meet family demands. Mothers may also be deemed less eligible for senior management positions due to relying on FWAs to satisfy family demands and resolve work–family conflicts. Yucel and Chung (2023) showed that the implementation of FWA policies escalate the level of work–family conflict for women, but not for men. As FWAs can disadvantage the career paths of working mothers, they may feel less inclined to use FWAs to cope with incompatible work and family roles and demands.

Compared to mother managers, father managers are often expected to fully commit to job tasks; thus, FWAs may not be associated with their work–family relations. However, this present study assumes that FWAs can help working fathers mitigate work–family conflicts, which in turn promotes a more positive perception of CEO gender equity and organizational commitment. The findings of existing studies have indicated that perceiving organizational support for families can reduce a father’s work–family conflict (Allard et al., 2011). Traditional gendered norms in the workplace assume that working fathers assume ‘breadwinning’ roles, devoting their time, attention, and motivation to work, while women take on homemaking roles (Cha & Kim, 2023).

Recently, the ideology of gender roles has been extended to promote equal participation by fathers and mothers (Huffman et al., 2014; Sievers, 2023). Increasing the involvement of fathers contributes to the well-being of the family by providing social and emotional support (Holmes et al., 2020). FWAs allow working fathers to share childcare responsibilities with their partners. Therefore, they help reduce work–family conflicts among working fathers, which can promote perceptions of CEO gender equity and increase organizational commitment. Ideal work schemes often require working fathers to prioritize job tasks and work overtime, while downplaying their family commitments (Huffman et al., 2014; Sievers, 2023). Thus, working fathers may find it challenging to use FWAs to balance work demands and family responsibilities.

As mentioned, the sample in this study consists of working mothers and fathers who hold professional and managerial positions, which tend to be well-paid and offer work–life balance, particularly as employees in these roles have the time, attention, and energy resources to promote this balance. Working fathers in these positions often take a keen interest in familial obligations and childcare (Holmes et al., 2020), motivated to balance their family commitments with their job performance and career advancement. Thus, father managers may rely on FWAs to maintain involvement in childcare and family roles by adjusting the time, space, and methods of their work.

**Hypothesis 3.**  *Gender moderates the interrelated relationships among FWAs, work–family conflict, CEO gender equality perceptions, and organizational commitment.*

**Figure 1:**
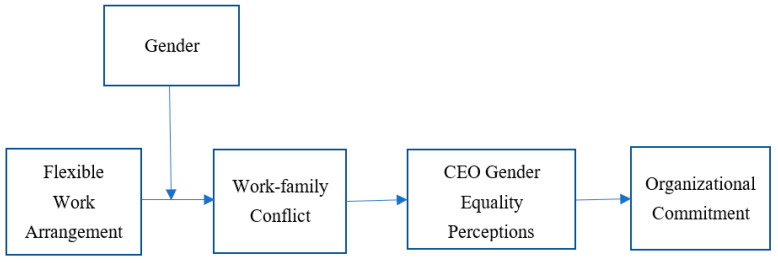
Research model.

Figure 1 depicts the serial moderated mediation relations of the study variables. Gender moderates the relationship between FWAs and work–family conflict. In turn, work–family conflict reduces managers’ perceptions of CEO gender equality, and CEO gender equality perception increases their organizational commitment.

## 3. Materials and Methods

### 3.1. Study Sample

This study’s sample draws upon the Korea Women Manager Panel (KWMP),^1^ conducted by the Korean Women’s Development Institute (KWDI), a research institute in Korea that promotes gender equality and family-supportive workplaces. As South Korea ranked at the bottom of countries worldwide for female representation in the labor market, the glass ceiling poses significant challenges for the career development of women workers (Lee et al., 2023). KWMP conducts an annual survey to track the career histories of female workers and identify the enablers and barriers that determine their career outcomes. It surveys the working conditions and family life of female and male managers, allowing for a gender comparison of career paths and work–family relations. The KWMP survey was structured at individual and organization levels. At the individual level, the KWMP survey examines female and male managers’ job attitudes, perceptions about organizational culture, quality of work and life, and participation in training and career development programs. At the organization level, the KWMP survey gathers data from human resource managers about sets of human resource policies and practices (e.g., recruitment, training, compensation, work–life quality, and promotion) relevant to managerial careers. KWDI provides the KWMP datasets on its website^2^.

The target of KWMP sampling process comprises male and female workers holding professional and managerial positions where they oversee at least 100 regular employees (Lee et al., 2023). The KWMP adopted a two-phase sampling technique to draw sampling for the current study. The first phase of sampling used stratified sampling techniques to take large-sized sampling for KWMP with the criteria of industry membership and company size. The first phase of sampling used a proportional allocation process to create sampling distribution. The first phase of sampling selected 3348 companies where the number of female managers is estimated to be 159,009. The second phase of sampling aims to secure 3500 female managers and 1500 male managers as the potential KWMP survey sample. Of 3340 companies selected in the first phase of sampling, the second phase of sampling drew 604 companies. From 604 companies, the KWMP survey selected 3500 female managers and 1511 male managers as survey respondents at the individual level. Concerning the organization level, the KWMP survey collected responses from one human resource manager at each company, which totals 604 human resource managers. Issues about sampling bias are raised as there is a sample size asymmetry between 3500 female and 1511 male managers. The KWMP survey used weighting factors to correct sampling biases from gender size difference^3^. Although there are concerns about sampling biases particularly from gender size asymmetry, the KWMP survey exerts efforts to mitigate estimation biases.

In 2020, KWMP began a large-scale questionnaire survey of the work and family life of 3500 female and 1511 male managers, including 640 human resource managers from 605 companies (Lee et al., 2023). The KWMP survey began in 2020 and had been conducted four times annually by 2023. To reinforce the causal relations of the research model, this current study utilizes variables from different years. The FWA variable is from the 2020 KWMP dataset, the work–family conflict variable is from the 2021 KWMP, and the CEO gender equality perceptions and organizational commitment variables are from the 2022 KWMP dataset. As child-rearing responsibilities comprise large shares of family work, parenthood significantly influences work–family relations. Thus, our study’s control manager sample is composed of mothers and fathers. The study sample includes 2345 mothers and fathers holding managerial positions across 469 companies. The FWA variable is constructed at the organization level, and the other variables (work–family conflict, CEO gender equality perceptions, and organizational commitment) are structured at the individual level. This study uses a multilevel structure to analyze the research model.

### 3.2. Variables

KWMP systematically made the questionnaire. The KWMP research team and expert panel (social science professors and government officials in the ministry of employment and labor) reviewed and improved its reliability and validity through several meetings and conferences. First, the KWMP research team conducted a literature review to generate questionnaire items, after which expert counsel reviewed the items and provided feedback to improve the quality of the survey. The KWMP research team then revised the questionnaire items by incorporating the feedback from the expert counsel (Lee et al., 2023). Some items were drawn from previous studies published in English. Korean scholars and researchers translated those questionnaire items published in international academic journals from English into Korean and used and validated them in Korean academic journals (Kim & Cha, 2010; Choi et al., 2014; Gwak & Choi, 2014).

#### 3.2.1. FWAs

Human resource officers were asked to report whether organizations use FWAs with ‘1’ as ‘use’ and ‘2’ as ‘no use.’ FWAs were measured using three items: (1) flex-time work, (2) alternative work schedules, and (3) homeworking/telecommuting. We dummy-coded each FWA measure as ‘1’ for ‘use’ and ‘0’ for ‘no use’ and summed the three FWA measures to assess use within the organization. We utilize the FWA measures reported by human resource officers in 2020.

#### 3.2.2. Work–Family Conflict

The KWMP draws work–family conflict items from Grzywacz and Marks (2000) (Kim & Cha, 2010; Choi et al., 2014). Negative work–family items include four items measured on a five-point Likert-scale: (1) “Your job reduces the effort you can give to activities at home;” (2) “Job worries or problems distract you when you are at home;” (3) “Personal or family worries and problems distract you when you are at work;” and (4) “Stress at home makes you irritable at work.” We averaged work–family conflict to assess work–family conflict reported in 2021.

#### 3.2.3. CEO Gender Equality Perceptions

CEO gender equality perception was measured with a five-point Likert scale using four items: (1) “Our CEO champions gender equality in the organization”; (2) “Our CEO treats employees equally regardless of their gender;” (3) “Our CEO trusts employees and perceives them as human capital;” (4) “Our CEO encourages employees to strike a balance between their work and family lives.” (Gwak & Choi, 2014). We then averaged the CEO gender equality perception items as reported by mother and father managers in 2022.

#### 3.2.4. Organizational Commitment

Organizational commitment items were drawn from N. J. Allen and Meyer (1990) and Mowday et al. (1979) and measured using three items: (1) “I feel as if this organization’s problems are my own;” (2) “I am proud to tell others that I am part of this organization;” and (3) “I think that I can become attached to this organization.” (Choi et al., 2014). Next, we averaged these three items as reported by mother and father managers in 2022.

#### 3.2.5. Gender

Manager gender is assigned a value of ‘1’ for men and ‘2’ for women. We dummy-coded manager gender as ‘1’ for mother managers and ‘0’ for father managers.

#### 3.2.6. Control Variables

The control variables are the ages and salaries of company managers, and the use of FWAs and easy to use FWAs. There can be contextual changes about the utilization of FWAs in 2020–2022. Once the COVID-19 pandemic began, FWAs had been actively initiated and disseminated across workplaces. Control variables include the use of FWAs and easy to use FWAs to reflect changes about FWA use. The use of FWAs variable was measured with whether company managers have chances to use FWAs. KWMP conducted the survey to assess the easy to use FWAs variable with a five-point Likert scale (1: very unlikely; 5: very likely). When company managers said ‘yes’ or ‘no’ about the “FWAs use” variable, they were required to respond to questions about the “easy use of FWAs”. The total number of managers who responded to “easy to use flextime”, “easy to use alternative work schedules”, and “easy to use homeworking/telecommuting” is 1504 managers, 1489 managers, and 1373 managers (Table A1 in Appendix A). When companies do not install FWAs, there are missing values in the “easy to FWAs use” variable. Missing values in the “easy to FWAs use” variable are coded into ‘zero’. Table A2 in Appendix A shows the descriptive statistics and one-way Anova analysis results. One-way Anova analysis showed significant differences for the use of FWAs and easy to use FWAs variables among three years, 2020, 2021, and 2022 (Table A3 in Appendix A). Controlling the use of FWAs and easy to use FWAs increases the validity of model analysis. All control variables, manager age, manager wage, the use of FWAs, and easy to use FWAs are constructed at the individual level.

## 4. Results

We use the multilevel macro process model 8 to examine the moderated mediation research model. First, we implement descriptive and correlation analyses. Second, we conduct a multilevel path analytic method to analyze the mediating sequential model.

### 4.1. Descriptive Statistics and Correlation Analyses

Table 1 presents descriptive statistics and correlation analyses results. Of the 2345 managers in the sample, 1593 are mothers and 752 are fathers^4^. The average age is 47.64 with a standard deviation of 6.94. Additionally, the average yearly wage is KRW 44,564 with a standard deviation of KRW 14,829. The average values of work–family conflict, CEO gender equality perceptions, and organizational commitment are 2.49, 3.45, and 3.79, respectively. CEO gender equality perceptions and organizational commitment variables are above moderate levels, and work–family conflict is below. Of the 469 companies, 156 companies use three FWA types, 95 companies use two types, 105 companies use one type, and 113 companies do not use FWAs. Specifically, 269 companies utilize flex-time work, 259 companies adopt alternative work schedules, and 235 companies use a homeworking/telecommuting program. Average values of the use of FWAs and easy to use FWAs are 0.66 and 2.02. Specifically, regarding the use of FWAs, 1437 managers do not have chances to use any FWAs; 476 managers used one FWAs; 267 managers used two FWAs; and 165 managers used three FWAs.

### 4.2. Multilevel Macro Process Model 8

With the multilevel macro process model 8, moderated mediation model analyses were conducted to analyze the mediating sequential relationships among FWAs, work–family conflict, CEO gender equality perceptions, and organizational commitment. Table 2 presents the statistical results of the multipath analysis regarding the mediation model. Notably, FWAs do not have any influence on work–family conflict (ß = 0.01, *p* > 0.10). Regarding gender, mother managers show higher levels of work–family conflict than father managers (ß = 0.17, *p* = 0.02). Gender and FWAs significantly interact in influencing work–family conflict (ß = 0.07, *p* = 0.02), while gender and FWAs fail to significantly interact when it comes to CEO gender equality (ß = −0.05, *p* > 0.10). Work–family conflict and gender are negatively related to CEO gender equality perceptions; father managers show increased levels compared to mother managers (work–family conflict: ß = −0.12, *p* = 0.00; gender: ß = −0.15, *p* = 0.05). FWAs, gender, the interaction between FWAs and gender, and the work–family conflict variables are not related to organizational commitment (FWAs: ß = −0.04, *p* > 0.05; gender: ß = 0.10, *p* > 0.10; interactions: ß = −0.01, *p* > 0.10; work–family conflict: ß = −0.02, *p* > 0.10). However, CEO gender equality perception is significantly related to organizational commitment (CEO gender equality perception: ß = 0.36, *p* = 0.00).

In sum, FWAs fail to reduce company managers’ work–family conflict. Thus, Hypothesis 1, which examines the effect of FWAs on the work–family conflict of company managers, is not supported. Furthermore, work–family conflict is negatively related to perceptions of CEO gender equality, and these perceptions are positively related to organizational commitment. Therefore, Hypothesis 2 is partially supported, as work–family conflict mediates the relationship between FWAs and CEO gender equality perceptions, and CEO gender equality perceptions promote organizational commitment. The interaction between gender and FWAs is positively related to work–family conflict; however, the interactive effect is not significantly related to CEO gender equality or organizational commitment. Thus, Hypothesis 3, which investigates the moderating role of gender in mediating the relationships among FWAs, work–family conflict, CEO gender equality perceptions, and organizational commitment, is partially supported.

Table 3 presents the direct and indirect effects of the mediation model. We conducted bootstrapping 500 times to calculate the direct and indirect effects of mediating relations. Regarding the direct and indirect effect of mediating relations for CEO gender equality perceptions, FWAs positively affect CEO gender equality (0.17), as the lower limit confidence interval (LLCI) and the upper limit confidence level (ULCI) do not include a “0” value (LLCI: 0.11 and ULCI: 0.22). The interaction between FWAs and the gender (−0.07) and work–family conflict (−0.19) variables negatively affect CEO gender equality perceptions, as a “0” value is not included in the interaction between LLCI and ULCI (interaction -> LLCI: −0.14 and ULCI: −0.01; work–family conflict -> LLCI: −0.23 and ULCI: −0.14). FWAs have marginal indirect effects (0.01) on CEO gender equality perceptions through work–family conflict, as LLCI is estimated to be −0.001. The interaction between gender and FWAs is negatively related to CEO perceptions of gender equality, mediated by work–family conflict (−0.02).

Regarding organizational commitment, the interaction between FWAs and gender fail to directly affect organizational commitment, as LLCI and ULCI include a “0” value (LLCI: −0.06 and ULCI: 0.04). The interactive effects of FWAs with gender indirectly affect organizational commitment through changes in CEO gender equality perceptions (FWAs: −0.01; interactions: 0.00), as LLCI and ULCI do not include a “0” value (FWAs -> LLCI: −0.02 and ULCI: −0.01; interactions -> LLCI: 0.00 and ULCI: 0.01). Work–family conflict and CEO gender equality perceptions have negative (−0.07) and positive effects (0.41) on organizational commitment, as there is no “0” value in the interaction between LLCI and ULCI (work–family conflict -> LLCI: −0.10 and ULCI: −0.03; CEO gender equality -> LLCI: 0.38 and ULCI: 0.44). Summarizing the direct and negative effects from the mediation model, the interaction between gender and FWAs influences CEO gender equality perceptions, mediated by work–family conflict. The interactive effect also indirectly impacts organizational commitment, mediated by perceptions of CEO gender equality. Work–family conflict negatively and indirectly affects organizational commitment through CEO gender equality perceptions. Calculating the indirect effects supports the serial mediating relations of FWAs, gender, work–family conflict, CEO gender equality perceptions, and organizational commitment.

### 4.3. Interaction Figures

**Figure 2:**
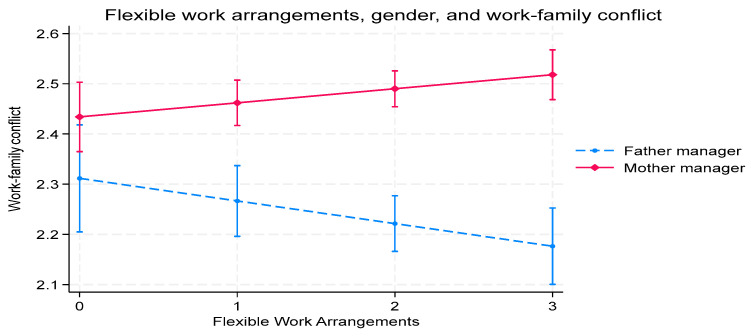
The impact of the interactions between manager gender and FWAs on work–family conflict.

**Figure 3:**
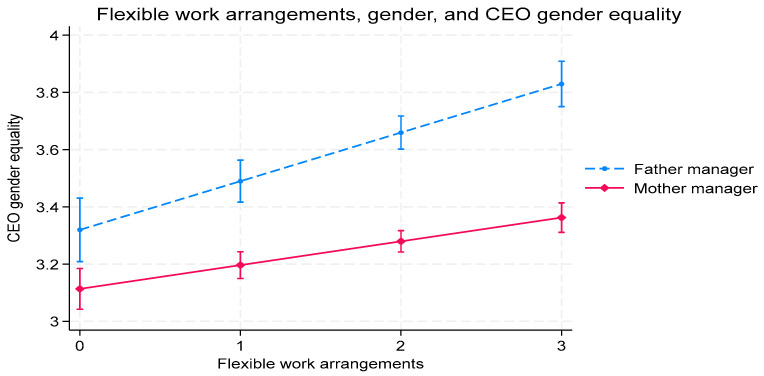
The impact of the interaction between manager gender and FWAs on CEO gender equality perceptions.

**Figure 4:**
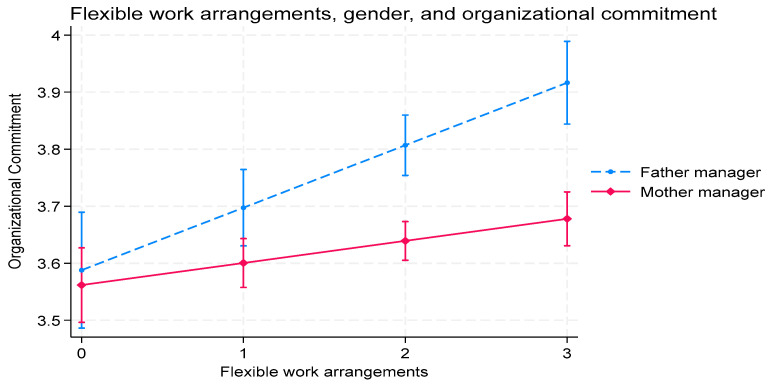
The impact of the interaction between manager gender and FWAs on organizational commitment.

We graph the interaction effects between FWAs and gender on work–family conflict (Figure 2), CEO gender equality perceptions (Figure 3), and organizational commitment (Figure 4).

Figure 2 presents the interactions between gender and FWAs. Father managers show reduced levels of work–family conflict when organizations make significant FWAs. In contrast, the work–family conflict of mother managers increases in correlation with FWA use. These findings suggest that father managers receive more benefits from FWAs than mother managers.

Figure 3 illustrates the relationship between gender and FWAs in influencing CEO gender equality. It reveals that the slope of father managers regarding FWAs and CEO gender equality increases more than that of mother managers as FWAs increase.

Figure 4 presents the interactive effects of gender and FWAs on organizational commitment, particularly when corporations are likelier to utilize FWAs. It also shows that father managers show greater organizational commitment than mother managers. The graph of the impact of the interaction between gender and FWAs on CEO gender equality indicates that FWAs foster more positive perceptions of CEOs and organizational commitment for father managers than mother managers.

### 4.4. Summary of Statistical Analyses 

Gender plays a moderating role in the relationship between FWAs and work–family conflict. Father managers experience less conflict while mother managers perceive more as FWAs increase. This conflict is negatively related to CEO gender equality perceptions, which in turn promotes organizational commitment of father managers. The mediating model presents significant indirect effects. In particular, the interaction between FWAs and gender indirectly affects CEO gender equality through changes in work–family conflict. Work–family conflict also indirectly affects organizational commitment, mediated by CEO gender equality perceptions. Therefore, FWAs, work–family conflict, CEO gender equality perceptions, and organizational commitment are sequentially linked, with work–family conflict and CEO gender equality perceptions playing mediating roles. Gender significantly affects the sequential relations of the mediation model: father managers are eligible for FWA benefits, while FWAs increase work–family conflict for mother managers. To increase the effectiveness of FWAs, it is crucial to examine gender roles to determine whether the permeability of the work–family boundary mitigates or exacerbates work–family conflict.

## 5. General Discussion

### 5.1. Research Findings and Discussion

This current study uses the multilevel macro process model 8 to examine FWAs, work–family conflict, CEO gender equality perceptions, and organizational commitment. Statistical analyses using the macro process model 8 reveal that FWAs mitigate work–family conflict, thereby enhancing perceptions of CEO gender equality and organizational commitment. With the use of FWAs, company managers can transfer roles and activities from work (family) into family (work) through mitigating boundaries between work and family domains (Oseghale et al., 2024). The mutual interference between the work and family domains exerts pressure on work–family compatibility, resulting in skeptical reactions toward CEOs and organizations. CEO perceptions of gender equality and organizational commitment are enhanced as FWAs decrease work–family conflict. To promote work-related attitudes, organizations must use FWAs to allow family boundaries to be permeable and flexible. Facilitating work–family transition using FWAs increases the recognition of a CEO’s role in adopting and extending work–family benefit programs and policies. FWAs support company managers to utilize behavioral strategies to negotiate work and family boundaries, which motivates them to reciprocate benefits from FWAs with positive perceptions and commitment toward the CEO and organization.

Furthermore, this study found that gender significantly affects the mediating relations among FWAs, work–family conflict, CEO gender equality perceptions, and organizational commitment. Specifically, FWAs are more beneficial to father managers than mother managers as they allow fathers to mitigate work–family conflict and promote CEO gender equality perceptions and organizational commitment. FWAs blur the boundaries between work and family, which may reinforce gendered assumptions about prioritizing work and family roles and activities (Yucel & Chung, 2023). Father managers may leverage FWAs effectively to respond to work–family conflict as they are presumed to be more actively involved in job task-related roles and activities (Yucel & Chung, 2023). Contrary to fathers, mother managers show higher levels of work–family conflict when companies increase FWAs. Family devotion often prevents mothers from maintaining their career progression, as traditional gender role beliefs urge them to engage in intensive parenting, causing them to forsake the idealized worker role and be pushed out due to overwork (Oh & Mun, 2022; Shin & Kim, 2023). Mother managers are vulnerable to pressures from satisfying work and family demands simultaneously. Therefore, for mother managers, increased FWAs can exacerbate stress due to the incompatible nature of the work and family domains; FWAs may result in adverse effects on the career progress of mother managers.

### 5.2. Theoretical Implications

We contribute to the existing literature by addressing several questions about the consequences of FWAs. First, this study develops a serial mediation model that extends the scope of exploring FWA effectiveness by examining gender roles, work–family conflict, CEO gender equality perceptions, and organizational commitment. Our model examines the extent to which father and mother managers perceive differences in the mediating processes of work–family conflict and CEO gender equity perceptions. It also examines the relationship between CEO gender equity perceptions and FWAs as well as organizational commitment. Articulating the process through which FWAs positively impact individuals and organizations, we provide supporting arguments for the use of FWAs. This approach advances understanding of the mechanisms through which FWAs shape managerial attitudes toward both work and family domains.

Second, the findings of this study extend the application scope of social exchange theory and boundary theory. When FWAs are available to company managers, these managers reciprocate the spatial and temporal workplace flexibility promoted through FWAs by enhancing their work-related attitudes and perceptions (Bayazit & Bayazit, 2019). Company managers define and manage the boundaries between work and family domains to exert control over work–family conflict. FWAs extends company managers’ discretions over work schedules and work locations to perform job tasks and work responsibilities, which blurs their work–family boundaries (Gajendran et al., 2024; Oseghale et al., 2024). The findings of this study reveal that flexibility in the workplace increases self-control over work schedule, location, and methods. It indicates that FWAs can be a valuable resource for supporting company managers in exerting more effective control over their allocation of time, energy, and attention to both their work and family domains, thereby confirming that social exchange theory and boundary theory are applicable to the organizational context of FWAs.

Third, this study contributes to gender and work–family research by revealing that FWAs yield asymmetric benefits based on gender. Father managers can leverage FWAs to enhance positive experiences in both work and home, whereas mother managers face stronger role expectations, intensifying work–family conflict and constraining career advancement. This demonstrates that gendered normative assumptions persist in the workplace and suggests that equal provision of FWAs does not necessarily guarantee equal outcomes.

Lastly, we aimed to advance FWA research by adopting a dataset spanning three years and a multilevel analysis method. This study’s dataset draws upon the Korean Women Manager Panel (KWMP), which covers 2020 to 2022 and includes variables selected from each year. Compared to cross-sectional studies, longitudinal designs provide a more rigorous framework for identifying the causal consequences of FWAs, particularly within the critical context of the COVID-19 pandemic. In addition, conducting a multilevel analysis more accurately accounts for within- and between-organization variances concerning FWAs and the related individual perceptions and attitudes. Future studies are needed to track the effects of the COVID-19 pandemic and its related changes in sets of HR practices and programs for several years, which will elucidate the impacts of the COVID-19 pandemic on organizations and workplaces.

### 5.3. Practical Implications

In terms of practical point of view, our findings offer implications about FWA effectiveness related to gender and the COVID-19 pandemic. First, this study demonstrated that FWAs are not merely a welfare program but a valuable strategic organizational resource that enhances perceptions of CEO’s fairness and parent managers’ organizational commitment. By granting managers greater autonomy over when, where, and how they perform their work, organizations can foster stronger psychological ownership and deeper engagement with organizational objectives. Thus, companies should treat FWAs as a core management tool that fosters organizational commitment and operate them systematically.

Second, the finding that FWAs mitigate work–family conflict, thereby increasing positive evaluations of CEOs’ gender equality perceptions, suggests that employees interpret flexible work policies as signals of leadership fairness and inclusivity. Therefore, CEOs and top management should actively and clearly communicate that the purpose of introducing FWAs is not merely to improve efficiency like productivity control, but to advance a management philosophy centered on trust, equity, and inclusivity (Shockley et al., 2021; Joo et al., 2024). This approach strengthens leadership credibility and helps foster managers’ organizational commitment.

Lastly, the COVID-19 pandemic accelerated the adoption and implementation of FWAs, as workspaces were closed down and company workers needed to work remotely to comply with social distancing guidelines (Toscano & Zappalá, 2024). In Korea, the usage of FWAs increased from 8% before the COVID-19 pandemic to more than 14% after the COVID-19 pandemic (Statistics Korea, 2025). Companies took actions to initiate and establish FWAs to cope with the COVID-19 pandemic lockdown (Toscano & Zappalá, 2024). While the COVID-19 outbreaks have receded, many workers still prefer to work remotely with the use of FWAs. Top managers express concerns about lower productivity and reduced levels of commitment to organizations and satisfied feelings about their jobs (Shockley et al., 2021; Gajendran et al., 2024). Existing studies examined the consequences of rapid and significant changes about work routines and practices caused by the adoption and use of FWAs after the COVID-19 pandemic lockdown ended (Shockley et al., 2021; Toscano & Zappalá, 2024). In particular, during the COVID-19 pandemic, mother managers were entitled to FWA benefits to cope with family roles and responsibilities more effectively. This study found that FWAs disproportionately benefit fathers over mothers in management roles, primarily due to persistent gender expectations regarding household and caregiving responsibilities during and even after the COVID-19 pandemic. Therefore, organizations must focus on implementing flexible work arrangements fairly, moving beyond the concept of ‘equal’ provision. This can include encouraging fathers to actively utilize flexible work arrangements for caregiving to normalize shared family responsibilities, while simultaneously providing tailored support for mothers—such as workload adjustments, mentoring, and career development assistance—to prevent reinforcing traditional gender stereotypes. Furthermore, companies should regularly review patterns of flexible work utilization and related career outcomes to identify and address gender gaps, ensuring these policies genuinely contribute to gender equality and fostering an inclusive organizational culture.

### 5.4. Limitations

This current study adopts a multilevel, multiyear structure and a sequential mediation model to extend the scope of research on FWAs and the related work–family conflict. Despite its strengths, we should consider certain limitations to better interpret the study results. First, the FWA variable is constructed using the sum of three dummy variables, flex-time work, alternative work schedules, and homeworking/telecommuting, to measure whether organizations use these FWA types. Future studies should use measures that evaluate the extent to which organizations adopt and utilize FWAs.

Second, there can be positive interdependence between work and family domains. FWAs provide discretion over where, when, and how managers perform their job tasks and work responsibilities, which reinforces positive relations between work and family. This may extend the implications of work–family relations when examining the effects of FWAs on the positive aspects of work–family.

Third, as the survey is sponsored by the Korean government, policies and regulations limited the financial resources available for generating, constructing, and utilizing questionnaire items. Future studies should construct variables that operationalize study concepts and fully capture the entirety of work–family conflict and organizational commitment measures.

Fourth, asymmetry exists in the sample size of mother and father managers, as the sample size for mothers is 1593 and 752 for fathers, raising questions about the appropriateness of the sample size in terms of gender comparison. Sample asymmetry may provide negative effects on the robustness and statistical power of gender comparisons. We conducted power analysis to confirm that the study sample size exceeds the threshold level of the sample size to satisfy statistical power (Cohen, 1988; Lix et al., 1996). Future studies are still needed to ensure symmetry in the sample size by gender as there could be concerns about the robustness of the study model.

Fifth, there could be gender differences in the accessibility and availability of FWAs for company managers. We conducted gender comparison analyses on the use of FWAs and easy to FWAs use variables (Appendix B). About “easy to FWAs use”, father managers find it easier to use FWAs compared to mother managers. About “the use of FWAs”, father managers can use FWAs slightly more than mother managers. The statistical results for gender differences in “the use of FWAs” and “easy to FWAs use” variables are consistent with the research model analysis: mother managers are less willing to use FWAs than fathers, even though mother managers experience more challenges from negative interactions between work and family domains.

Sixth, Anova analysis shows that there are significant differences between the “FWAs use” and “easy to FWAs use” variables among the three years (Table A2 in Appendix A). The COVID-19 pandemic was reported in December 2019; social distancing guidelines were enacted in 2020 to ensure workers’ safety during the COVID-19 pandemic; and the COVID-19 pandemic ended in May 2023. This study explores the effects of FWA use on managers’ work attitudes during and after the COVID-19 pandemic over the three-year period from 2021 to 2023. We should consider the possibility that FWA use and its related work attitudes experience changes during and after the COVID-19 pandemic, which influences the comparability of the variables across years.

Finally, the sample of the current study is restricted to mothers and fathers who hold professional and managerial positions in companies with more than 100 workers. But in Korean workplaces, the demographics and job status of workers are different from the current study sample, such as single, married without children, temporary, and lower-wage workers. For the current study sample, mother and father managers possess resources and motivations to respond to negative interdependence between work and family domains. Many Korean workers have fewer resources to exert control over work schedules to ameliorate work–family conflict. Interpreting and applying the present study’s research implications into FWA planning and implementation should define who are eligible for FWAs and the purposes of using FWAs. Companies can categorize female workers into a couple of groups in the use of FWAs. To promote the quality of work–family balance for professional and managerial mothers, companies should consider significant influences from traditional concepts about gender and work and family roles and activities. For female workers who are single, married without children, or have temporary or low-paying positions, FWA support should be provided to them to address work–family conflict and enhance the quality of work–family life.

### 5.5. Conclusions

This study’s findings reveal that FWAs shape and establish work–family boundaries, allowing them to be permeable and flexible, which alleviates work–family conflict and fosters positive attitudes toward CEOs and organizations. The study framework advances the knowledge that guides the process through which FWAs positively impact managers. The statistical results show that FWA effectiveness is more prominent for father managers, as they are less vulnerable to gendered norms regarding work and family life. Contrary to expectations, FWAs have a limited contribution for mother managers. Mothers are often normatively pressured to devote themselves to their families and neglect their career paths. Thus, FWAs can reinforce gender stereotypes about mother managers prioritizing family life over company work. Mothers are less motivated to use FWAs as they fear career disadvantages from gender-stereotyped assumptions about work and family life. In contrast, FWA benefits are more accessible to fathers, as they are less constrained by stereotypes about work–family relations from FWAs. Thus, it is pivotal to accommodate gender roles to promote FWA effectiveness. We hope that this study provides evidence for FWA planning and implementation to enhance the acquisition, retention, and development of human capital by alleviating work and family incompatibility.

## Figures and Tables

**Table 1:** Descriptive and correlation analyses.

Individual Level ^1^										
Variables	Mean	Standard Deviation	1	2	3	4	5	6	7	8
1. Age	47.64	6.94								
2. Wage (yearly)	KRW 44,564	KRW 14,829	0.15 *							
3. The use of FWAs	0.66	1.00	−0.10	0.24						
4. Easy to FWAs use	2.02	1.60	0.03	0.37	0.55					
5. Gender	0.68	0.46	−0.02	−0.24 *	−0.07	−0.12				
6. Work–family conflict	2.49	0.89	−0.28 *	−0.16 *	0.30	0.07	0.20 *			
7. CEO gender equality perceptions	3.45	0.94	0.15 *	0.19 *	0.01	0.28	−0.18 *	−0.21 *		
8. Organizational commitment	3.79	0.82	0.22 *	0.24 *	0.07	0.18	−0.09 *	−0.17 *	0.48 *	
Organization level ^2^										
7. FWAs	1.62	1.17	0.03	0.41 *	0.46 *	0.72 *	−0.05	0.05	0.17 *	0.09

^1^ 2345 managers; ^2^ 469 companies; * *p* < 0.01.

**Table 2:** Multilevel macro process model 8 analyses.

Dependent Variables	Variables	Coefficient	Standard Error	z	*p*	LLCI	ULCI
Work–family conflict	Age	−1.58	0.12	−12.75	0.00	−1.82	−1.34
	Wage	−0.19	0.06	−3.04	0.00	−0.31	−0.07
	The use of FWAs	0.03	0.02	1.59	0.11	−0.01	0.07
	Easy to FWAs use	−0.04	0.01	−2.60	0.01	−0.07	−0.01
	Gender × FWAs	0.07	0.03	2.23	0.02	0.01	0.14
	FWAs	0.01	0.03	0.42	0.67	−0.05	0.07
	Gender	0.17	0.07	2.29	0.02	0.02	0.32
	Constant	10.05	0.65	15.38	0.00	8.76	11.33
CEO gender equality perceptions	Age	0.57	0.13	4.32	0.00	0.31	0.83
	Wage	0.08	0.06	1.30	0.19	−0.04	0.21
	The use of FWAs	−0.04	0.02	−1.71	0.08	−0.08	0.00
	Easy to FWAs use	0.22	0.01	13.24	0.00	0.18	0.25
	Work–family conflict	−0.12	0.02	−5.99	0.00	−0.16	−0.08
	Gender × FWAs	−0.05	0.03	−1.50	0.13	−0.12	0.01
	FWAs	−0.04	0.03	−1.23	0.22	−0.11	0.02
	Gender	−0.15	0.08	−1.95	0.05	−0.31	0.00
	Constant	0.63	0.70	0.90	0.37	−0.74	2.01
Organizational commitment	Age	0.72	0.11	6.65	0.00	0.51	0.93
	Wage	0.35	0.05	6.67	0.00	0.25	0.46
	The use of FWAs	−0.02	0.02	−1.01	0.31	−0.05	0.02
	Easy to FWAs use	0.05	0.01	3.51	0.00	0.02	0.08
	CEO gender equality	0.36	0.02	20.97	0.00	0.33	0.39
	Work–family conflict	−0.02	0.02	−1.24	0.21	−0.05	0.01
	Gender × FWAs	−0.01	0.03	−0.54	0.59	−0.07	0.04
	FWAs	−0.04	0.03	−1.66	0.09	−0.10	0.01
	Gender	0.10	0.06	1.58	0.11	−0.02	0.23
	Constant	−3.31	0.57	−5.74	0.00	−4.44	−2.18

The FWAs variable is measured using 469 companies, and the other variables are measured using 2345 company managers.

**Table 3:** Direct and indirect mediating effects.

Effect	Independent Variable	Mediating Variable	Dependent Variable	Effect	Standard Error	LLCI	ULCI
Direct	FWAs	-	CEO gender equality perceptions	0.17	0.03	0.11	0.22
Indirect		Work–family conflict		0.01	0.00	−0.00	0.02
Direct	Gender × FWAs	-		−0.07	0.03	−0.14	−0.01
Indirect		Work–family conflict		−0.02	0.00	−0.03	−0.00
Direct	Work–family Conflict			−0.19	0.02	−0.23	−0.14
Direct	FWAs	-	Organizational commitment	0.03	0.02	−0.01	0.07
Indirect		CEO gender equality		−0.01	0.00	−0.02	−0.01
Direct	Gender × FWAs	-		−0.01	0.03	−0.06	0.04
Indirect		CEO gender equality		0.00	0.00	0.00	0.01
Direct	Work–family conflict	-		−0.07	0.02	−0.10	−0.03
Indirect		CEO gender equality		−0.08	0.01	−0.10	−0.05
Direct	CEO gender equality			0.41	0.01	0.38	0.44

## Data Availability

The raw data supporting the conclusions of this article will be made available by the authors upon request.

## Notes

1 KWMP is a panel dataset that longitudinally tracks the career paths of both female and male managers. Its primary objective is to examine the representation of women in management positions and assess the current state of gender diversity in Korean corporations. By collecting a wide range of information, including demographic characteristics, workplace data, and family-related variables, from male and female managers, KWMP enables researchers to conduct gender-based comparisons of managerial careers.

2 See https://gsis.kwdi.re.kr/kwmp/data/eng/downloadView.do (accessed on 15 July 2025).

3 weighting factors = $\frac{population\;size\;in\;the\;first\;phase}{sample\;size\;in\;the\;second\;phase\;}*\frac{population\;size\;in\;the\;second\;phase}{sample\;size\;in\;the\;second\;phase}*\frac{1}{female\;and\;male\;managers\;sampling\;proportion}$

4 Regarding the number of children, 781 managers have one child (33.3%), 1376 have two children (58.68%), 176 have three children (7.51%), and 12 have four children (0.5%).

## Appendix A

**Table A1:** Proportions and sample size for use of FWAs and easy to FWAs use by specific programs.

FWAs	FWAs Use			Easy to Use FWAs					Sample Size
	Use	No Use	Not Available	1	2	3	4	5	
Flex-time work	474	888	142	65	122	310	378	629	1504
	(31%)	(59%)	(9%)	(4.32%)	(8.11%)	(20.61%)	(25.13%)	(41.82%)	managers
Alternative work scheduling	327	966	196	80	178	337	346	548	1489
	(22%)	(65%)	(13%)	(5.37%)	(11.95%)	(22.63%)	(23.24%)	(36.80%)	managers
Homeworking/telecommuting	548	673	152	128	224	331	291	399	1373
	(40%)	(49%)	(11%)	(9.32%)	(16.31%)	(24.11%)	(21.19%)	(29.06%)	managers

**Table A2:** Descriptive statistics for use of FWAs and easy to FWAs use.

Variable	Year	Mean	Standard Deviation
The use of FWAs	2020	0.59	0.85
	2021	0.66	0.89
	2022	0.66	1.00
Easy to use FWAs	2020	2.90	1.88
	2021	3.01	1.85
	2022	2.82	1.77

**Table A3:** One-way Anova analysis for use of FWAs and easy to FWAs use by years.

Variables	Source	SS	df	MS	F	*p*-Value
The use of FWAs	Between groups	9.06	2	4.53	6.05	0.00
	Within groups	5270.86	7032	0.75		
	Total	5279.92	7034	0.75		
Easy to use FWAs	Between groups	46.14	2	23.07	7.31	0.00
	Within groups	22,184.18	7030	3.15		
	Total	22,230.33	7032	3.16		

## Appendix B

**Table A4:** Analysis about gender difference in FWA use and easy to FWAs use among years.

Year	Variable	Gender	Mean	Standard Deviation	t-Value	*p*-Value
2020	FWAs use	Mother	0.58	0.85	1.12	0.26
		Father	0.62	0.85		
	Easy to FWAs use	Mother	2.76	1.88	5.30	0.00
		Father	3.19	1.84		
2021	FWAs use	Mother	0.64	0.89	1.50	0.13
		Father	0.70	0.89		
	Easy to FWAs use	Mother	2.84	1.88	6.45	0.00
		Father	3.36	1.75		
2022	FWAs use	Mother	0.62	0.97	3.38	0.00
		Father	0.77	1.07		
	Easy to FWAs use	Mother	2.67	1.78	5.81	0.00
		Father	3.12	1.72		
